# Supplementary material for: Boost the resilience of protected areas to shocks by reducing their dependency on tourism
Source: PLoS One. 2023 Apr 13;18(4):e0278591. doi: 10.1371/journal.pone.0278591 (PMC10101445; doi:10.1371/journal.pone.0278591)
Supplement: S1 File — Information, data and sources considered to document Madagascar’s revenues from nature-based tourism and costs of management for conservation: The 43 Protected Areas considered in this study and their suitability for nature-based tourism (Table A). Tickets sold by MNP to visit PAs (terrestrial and marine) from 2017 to 2021 (Table B). Entrance fees rate for PAs not aligning to the rate of MGA 45, 000 starting in 2016 (Table C). Evolution of the top 10 PAs visited over 30 years and from 2011 to 2019 according to their main appeal (Table D). Top 10 PAs in number of tickets sold VS. Top 10 PAs in revenue generated from 2017 to 2019, according to their main appeal (Table E). Conservation Trust Funds: The Foundation for Protected Areas and Biodiversity of Madagascar (Fondation pour les Aires Protégées et la Biodiversité de Madagascar FAPBM) (Box A). (PDF) [file pone.0278591.s001.pdf]

# Boost the resilience of protected areas to shocks by reducing their dependency on tourism

## Supporting Information

**Table A. The 43 Protected Areas considered in this study and their suitability for ecotourism. (ordered chronologically based on date of initial decree; NP = National Park; SR = Special Reserve; SNR = Strict Nature Reserve)**

| Name | IUCN | Size (ha) | Created | Accessibility | Attractions | Ecotourism |
|---|---|---|---|---|---|---|
| Andringitra NP[1] | II | 31,160 | 31/12/1927 | RN7 459 km from TNR, secondary road 47–90 km, 2 days | Landscape, mountain, forest | suitable |
| Ankarafantsika NP[2] | II | 136,513 | 31/12/1927 | RN4, 450 km from Antananarivo and 115 km from Mahajanga, 1 day | Forest biodiversity, wetland, geoscape, cultural sites | suitable |
| Tsingy de Bemaraha NP[3] | II | 157,710 | 31/12/1927 | Flight + 4*4 vehicle (8 hours), 800 km from Tana, 2—3 days | Landscape, biodiversity, cultural sites | suitable |
| Betampona SNR[4] | Ia | 2,228 | 31/12/1927 | | | ./. |
| Lokobe NP[5] | II | 862 | 31/12/1927 | Flight to Nosy Be, road or boat | Forest | suitable |
| Tsingy de Namoroka NP[6] | II | 22,227 | 31/12/1927 | RN4 552 km, ferry + secondary road RNT19 220 km, 3 days | Geoscape | potential |
| Tsaratanàna SNR[7] | Ia | 108,610 | 31/12/1927 | | | ./. |

[1] https://www.parcs-madagascar.com/parcs/andringitra.php
[2] https://www.parcs-madagascar.com/parcs/ankarafantsika.php
[3] https://www.parcs-madagascar.com/parcs/bemaraha.php
[4] https://www.parcs-madagascar.com/parcs/betampona.php
[5] https://www.parcs-madagascar.com/parcs/lokobe.php
[6] https://www.parcs-madagascar.com/parcs/baie_de_baly.php
[7] https://www.parcs-madagascar.com/parcs/tsaratanana.php

| Name | IUCN | Size (ha) | Created | Accessibility | Attractions | Ecotourism |
|---|---|---|---|---|---|---|
| Tsimanampesotse NP[8] | II | 202,525 | 31/12/1927 | Flight + boat + 4*4 vehicle, 2 – 3 days | Spiny forest, biodiversity, wetland, avens | suitable |
| Zahamena NP[9] | II | 64,935 | 31/12/1927 | RN2 to Moramanga, RN44, 348 km total from Tana, 2 – 3 days | Forest biodiversity | potential |
| Andohahela NP[10] | II | 76,140 | 11/06/1939 | Flight, RN 13, 1 – 2 day(s) | Diversity of vegetation types from humid to spiny | suitable |
| Marojejy NP[11] | II | 60,050 | 03/01/1952 | Flight, vehicle, 2 days | Forest biodiversity, mountain | suitable |
| Analamerana SR[12] | IV | 34,700 | 20/02/1956 | Flight, 4*4 vehicle, 2 days | Forest biodiversity, geoscape | potential |
| Ankarana SR[13] | IV | 18,225 | 20/02/1956 | Flight, 4*4 vehicle, 2 days | Forest biodiversity, geoscape, wetland | suitable |
| Manongarivo SR[14] | IV | 51,568 | 20/02/1956 | Flight, RN6 230 km, RN4 42 km, 4*4 vehicle, long walk, 3 – 4 days | Forest biodiversity | ./. |
| Marotandrano SR[15] | IV | 42,200 | 20/02/1956 | 1200 km from Tana: RN6 950 km, 4*4 vehicle RN 32,32A 250 km \| 2 – 3 days | Forest biodiversity | ./. |
| Ambatovaky SR[16] | IV | 78,139 | 28/10/1958 | RN2, RN5, ferry, walking, canoeing 4 – 5 days | Forest biodiversity | ./. |
| Andranomena SR[17] | IV | 8,170 | 28/10/1958 | Flight to Morondava + 4*4 vehicle (1 hour); 700 km from Tana, 2 days | Dry forest biodiversity | potential |
| Anjanaharibe-Sud SR[18] | IV | 26,903 | 28/10/1958 | Flight to Sambava, vehicle, walking for several hours, 2 days | Humid forest biodiversity | ./. |

[8] https://www.parcs-madagascar.com/parcs/tsimanapesotse.php
[9] https://www.parcs-madagascar.com/parcs/zahamena.php
[10] https://www.parcs-madagascar.com/parcs/andohahela.php
[11] https://www.parcs-madagascar.com/parcs/marojejy.php
[12] https://www.parcs-madagascar.com/parcs/analamerana.php
[13] https://www.parcs-madagascar.com/parcs/ankarana.php
[14] https://www.parcs-madagascar.com/parcs/manongarivo.php
[15] https://www.parcs-madagascar.com/parcs/marotandrano.php
[16] https://www.parcs-madagascar.com/parcs/ambatovaky.php
[17] https://www.parcs-madagascar.com/parcs/kirindy-mite.php
[18] https://www.parcs-madagascar.com/parcs/anjanaharibe %20sud.php

| Name | IUCN | Size (ha) | Created | Accessibility | Attractions | Ecotourism |
|---|---|---|---|---|---|---|
| Mangerivola SR[19] | IV | 11,900 | 28/10/1958 | RN2, motorbike, long walks, 2 days | Humid forest biodiversity | ./. |
| Montagne d'Ambre NP[20] | II | 30,538 | 28/10/1958 | Flight, vehicle, 2 days | Humid forest biodiversity, landscape, wetlands | suitable |
| Kalambatritra SR[21] | IV | 28,255 | 24/04/1959 | 765 km including secondary road, walking 35km, 3 - days | Humid forest biodiversity | ./. |
| Isalo NP[22] | II | 81,540 | 19/07/1962 | RN7, 2 days | Geoscape | suitable |
| Cap Sainte Marie SR[23] | IV | 3,610 | 29/10/1962 | Flight, 4*4 vehicle, 2 days | Landscape, vegetation type | suitable |
| Manombo SR[24] | IV | 5,320 | 05/12/1962 | RN7+RN25 708 km, 2 days | Littoral forest | potential |
| Pic d'Ivohibe SR[25] | IV | 3,453 | 16/09/1964 | RN7, secondary road, 3 days | Humid forest, mountain | ./. |
| Nosy Mangabe NP[26] | II | 729 | 14/12/1965 | Flight, boat, 2 days | Humid forest, landscape, seascape | suitable |
| Analamazaotra NP[27] | II | 874 | 21/07/1970 | RN2, 4 hours | Humid forest biodiversity | suitable |
| Ambohitantely SR[28] | IV | 5,600 | 12/02/1982 | RN4, 4 hours | Forest fragments | potential |
| Bezà-Mahafaly SR[29] | IV | 4,200 | 04/06/1986 | Flight, 4*4 vehicle, 2 – 3 days | Spiny forest biodiversity, ripisylve | potential |
| Mantadia NP[30] | II | 15,480 | 11/01/1989 | RN2, 4*4 vehicle xx km, 5 hours | Humid forest biodiversity | suitable |
| Mananara-Nord NP[31] | II | 24,000 | 25/07/1989 | Flight, RN5 or boat, long walk, 2 – 4 days | Humid forest biodiversity, marine | potential |
| Ranomafana NP[32] | II | 43,550 | 07/05/1991 | RN7, RN25, 1 long day | Humid forest biodiversity | suitable |

[19] https://www.parcs-madagascar.com/parcs/mangerivola.php
[20] https://www.parcs-madagascar.com/parcs/montagne %20d %20ambre.php
[21] https://www.parcs-madagascar.com/parcs/kalambatritra.php
[22] https://www.parcs-madagascar.com/parcs/isalo.php
[23] https://www.parcs-madagascar.com/parcs/ste-marie.php
[24] https://www.parcs-madagascar.com/parcs/manombo.php
[25] https://www.parcs-madagascar.com/parcs/ivohibe.php
[26] https://www.parcs-madagascar.com/parcs/masoala.php
[27] https://www.parcs-madagascar.com/parcs/analamazaotra.php
[28] https://www.parcs-madagascar.com/parcs/ambohitantely.php
[29] https://www.parcs-madagascar.com/parcs/beza %20mahafaly.php
[30] https://www.parcs-madagascar.com/parcs/mantadia.php
[31] https://www.parcs-madagascar.com/parcs/mananara-nord.php
[32] https://www.parcs-madagascar.com/parcs/ranomafana.php

| Name | IUCN | Size (ha) | Created | Accessibility | Attractions | Ecotourism |
|---|---|---|---|---|---|---|
| Masoala NP[33] | II | 230,000 | 02/03/1997 | Flight, boat, 2 days | Humid forest biodiversity, landscape, seascape | suitable |
| Baie de Baly NP[34] | II | 57,418 | 18/12/1997 | RN4 552 km, ferry + secondary road RNT 19 km, 2 – 3 days | Dry forest biodiversity, seascape | ./. |
| Befotaka / Midongy NP[35] | II | 192,198 | 18/12/1997 | RN7+RN25 872 km, motorbike, 4 days | Humid forest biodiversity | ./. |
| Kirindy Mité NP[36] | II | 156,350 | 18/12/1997 | Flight, 4*4 vehicle or boat, 1 – 2 day(s) | Dry forest biodiversity, wetlands, seascape | suitable |
| Zombitse-Vohibasia NP[37] | II | 36,803 | 18/12/1997 | RN7, 2 days | Dry forest biodiversity | suitable |
| Sahamalaza/Iles Radama NP[38] | II | 26,035 | 19/03/2007 | RN4+RN6, boat, 2 – 3 days | Dry forest biodiversity, seascape | potential |
| Mikea NP[39] | II | 184,630 | 06/07/2011 | Flight, 4*4 vehicle, 2 – 3 days | Dry spiny forest biodiversity, seascape | ./. |
| Nosy Hara NP[40] | II | 125,471 | 06/09/2011 | Flight, boat, 2 days | Seascape, landscape | potential |
| Nosy Tanikely NP[41] | II | 180 | 06/09/2011 | Flight, boat, 2 days | Seascape, seaside | suitable |
| Marolambo NP[42] | II | 95,063 | 21/04/2015 | RN7, walk or motorbike, 2 days | Humid forest biodiversity, landscape | ./. |
| Nosy Ve Androka NP[43] | II | 91,445 | 23/07/2015 | Flight, boat, 2 days | Seascape | potential |

[33] https://www.parcs-madagascar.com/parcs/masoala.php
[34] https://www.parcs-madagascar.com/parcs/baie_de_baly.php
[35] https://www.parcs-madagascar.com/parcs/midongy %20du %20sud.php
[36] https://www.parcs-madagascar.com/parcs/kirindy-mite.php
[37] https://www.parcs-madagascar.com/parcs/zombitse.php
[38] https://www.parcs-madagascar.com/parcs/sahamalaza.php
[39] https://www.parcs-madagascar.com/parcs/mikea.php
[40] https://www.parcs-madagascar.com/parcs/nosy %20hara.php
[41] https://www.parcs-madagascar.com/parcs/nosy-tanihely.php
[42] https://www.parcs-madagascar.com/parcs/marolambo.php
[43] https://www.parcs-madagascar.com/parcs/nosy-ve-androka.php

**Table B. Tickets sold by MNP to visit PAs (terrestrial and marine) from 2017 to 2021.**

| Categories \| years | 2017 | 2018 | 2019 | 2020 | 2021 |
|---|---|---|---|---|---|
| Adult foreign | 140,391 | 150,901 | 166,907 | 12,658 | 3,297 |
| Adult Malagasy | 40,675 | 43,750 | 54,061 | 10,835 | 24,159 |
| Child (foreign) | 4,937 | 4,475 | 4,876 | 475 | 305 |
| Child (national) | 3,246 | 3,856 | 4,097 | 1,206 | 3,130 |
| Total | 189,249 | 202,982 | 229,941 | 25,174 | 30,891 |
| Total foreigners | 145,328<br>77 % | 155,376<br>77 % | 171,783<br>75 % | 13,133<br>52 % | 3,602<br>12 % |
| Total nationals | 43,921<br>23 % | 47,606<br>23 % | 58,158<br>25 % | 12,041<br>48 % | 27,289<br>88 % |

**Table C. Entrance fees rate for PAs not aligning to the rate of MGA 45,000 starting in 2016. (eq. US$ 13.38 in 2016 for Malagasy Ariary MGA)**

| PAs | Rate in MGA | US$ equivalent in 2016 |
|---|---|---|
| Isalo NP | 65,000 | 19.33 |
| Ranomafana NP | 55,000 | 16.36 |
| Nosy Tanihely NP | 20,000 | 5.95 |
| Montagne d'Ambre NP | 55,000 | 16.36 |
| Bemaraha NP | 55,000 | 16.36 |
| Ankarana SR | 65,000 | 19.33 |
| Ankarafantsika NP | 55,000 | 16.36 |
| Lokobe NP | 55,000 | 16.36 |
| Andohahela NP | 55,000 | 16.36 |
| Nosy Hara NP | 55,000 | 16.36 |

**Table D. Evolution of the top 10 PAs visited over 30 years and from 2011 to 2019 according to their main appeal.**

| Top 10 PAs 1992–2021 | | Top 10 PAs 2011–2019 | |
|---|---|---|---|
| **PAs** | **% *** | **PAs** | **% *** |
| Isalo | 21.1 % | Nosy Tanikely | 18.7 % |
| Mantadia | 19.9 % | Isalo | 17.8 % |
| Analamazaotra | | Mantadia | 16.4 % |
| Ranomafana | 14.3 % | Analamazaotra | |
| Nosy Tanikely | 10.6 % | Ranomafana | 13.1 % |
| Montagne d'Ambre | 9.3 % | Bemaraha | 7.8 % |
| Bemaraha | 6.3 % | Montagne d'Ambre | 7.1 % |
| Ankarana | 6.0 % | Ankarana | 5.8 % |
| Ankarafantsika | 3.1 % | Ankarafantsika | 2.5 % |
| Masoala | 1.9 % | Lokobe | 2.3 % |

*number of visits in the PA / total visits in the network of 43 PAs; green for terrestrial biodiversity, blue for marine PAs, grey for geosites, i.e., geological or geomorphological features; yellow for biodiversity and geosite

**Table E. Top 10 PAs in number of tickets sold VS. top 10 PAs in revenues generated from 2017 to 2019, according to their main appeal.**

| | 2017 | | 2018 | | 2019 | | 2017–2019 | |
|---|---|---|---|---|---|---|---|---|
| **Top 10 PAs (tickets)** | **Rank** | **% *** | **Rank** | **% *** | **Rank** | **% *** | **Rank** | **% **** |
| Nosy Tanikely | 1 | 20.0 % | 1 | 21.7 % | 1 | 22.0 % | 1 | 21.3 % |
| Isalo | 2 | 17.1 % | 3 | 16.1 % | 2 | 16.8 % | 2 | 16.7 % |
| Ranomafana | 3 | 16.3 % | 2 | 16.6 % | 3 | 16.0 % | 3 | 16.3 % |
| Mantadia, Analamazaotra | 4 | 13.5 % | 4 | 11.6 % | 4 | 11.9 % | 4 | 12.3 % |
| Bemaraha | 5 | 11.1 % | 6 | 7.9 % | 5 | 9.4 % | 5 | 9.4 % |
| Montagne d'Ambre | 6 | 5.2 % | 5 | 8.6 % | 6 | 6.5 % | 6 | 6.8 % |
| Ankarana | 7 | 3.9 % | 7 | 4.1 % | 7 | 4.3 % | 7 | 4.1 % |
| Zombitse-Vohibasia | 10 | 1.8 % | 8 | 2.5 % | 8 | 2.4 % | 8 | 2.3 % |
| Ankarafantsika | 8 | 2.3 % | 9 | 2.3 % | 9 | 1.9 % | 9 | 2.2 % |
| Masoala, Nosy Mangabe | 9 | 1.9 % | 10 | 1.8 % | 10 | 1.8 % | 10 | 1.8 % |
| Totals | | 93.2 % | | 93.2 % | | 92.9 % | | 93.1 % |
| **Top 10 PAs (revenues)** | **Rank** | **% *** | **Rank** | **% *** | **Rank** | **% *** | **Rank** | **% *** |
| Isalo | 1 | 23.5 % | 1 | 22.2 % | 1 | 23.2 % | 1 | 22.9 % |
| Mantadia, Analamazaotra | 2 | 16.4 % | 3 | 14.6 % | 2 | 14.9 % | 2 | 15.2 % |
| Ranomafana | 3 | 14.9 % | 2 | 15.3 % | 3 | 14.8 % | 3 | 15.0 % |
| Bemaraha | 4 | 13.5 % | 5 | 10.4 % | 4 | 12.1 % | 4 | 12.0 % |
| Nosy Tanikely | 5 | 8.3 % | 6 | 9.4 % | 5 | 9.9 % | 5 | 9.2 % |
| Montagne d'Ambre | 6 | 6.6 % | 4 | 10.5 % | 6 | 7.1 % | 6 | 8.0 % |
| Ankarana | 7 | 5.8 % | 7 | 6.2 % | 7 | 6.7 % | 7 | 6.3 % |
| Zombitse-Vohibasia | 9 | 2.2 % | 8 | 3.1 % | 8 | 2.9 % | 8 | 2.8 % |
| Masoala, Nosy Mangabe | 10 | 2.1 % | 10 | 2.0 % | 10 | 2.0 % | 9 | 2.0 % |
| Ankarafantsika | 8 | 2.5 % | 9 | 2.4 % | 9 | 2.0 % | 10 | 2.3 % |
| Totals | | 95.7 % | | 96.1 % | | 95.7 % | | 95.8 % |

*number of visits or revenues in the PA / total visits or total revenue in the network of 43 PAs; **ranked according to the 2017–2019 totals; green for terrestrial biodiversity, blue for marine PAs, grey for geosites, i.e., geological or geomorphological features; yellow for biodiversity and geosite; Mantadia and Analamazaotra NPs, belong to the same management unit, and tickets sold are for both PAs, the same applies to Masoala and Nosy Mangabe.

**Box A. Conservation Trust Funds: The Foundation for Protected Areas and Biodiversity of Madagascar (Fondation pour les Aires Protégées et la Biodiversité de Madagascar FAPBM)**[1]

*Sustainable funding of protected areas*

The Fund for Protected Areas and Biodiversity of Madagascar (FAPBM) is a private trust created in 2005 as a mechanism to sustainably fund conservation and biodiversity within PAs. As of 2018, FAPBM ranked among the top 10 % of global conservation trust funds (CTE) in terms highest amounts of capital holdings at US$ 69 million. The main capital of the foundation consists of endowment funds, but other funding methods are also employed—e.g., sinking funds—and debt-for-nature swaps[2]. To maintain its capital value, FAPBM needs to reinvest part of its annual income equal to inflation in its endowment or sinking funds. Its expenditure is 3–4 % per year, moving on an average over three years, i.e., within the range of most conservation trust funds[2]. The revenues are distributed as grants for the management of PAs, most of the time according to conventions between the funding agencies and FAPBM. One example of this is the endowment funds provided by the German state-owned investment and development bank KfW, (i) in 2016, 22 million USD was given to benefit only MNP, while (ii) in 2021, 60 % of 45.7 million euros will benefit MNP and the remaining 40 % will fund so-called new PAs managed by NGOs (IUCN categories V and VI).

*The foundation has several mechanisms in place to receive funding:*

- Endowment funds (*fonds de dotation au capital*): FAPBM began receiving funds in 2005. These funds have increased over the years to ca. 139 million USD at the end of 2021. Only the interest or investment income are spent: this started in 2007 for FAPBM. The annual interest obtained on the capital endowment is ca. 3.25 % per year. Endowment funds are designed to operate in perpetuity with no fixed term.
- Debt swap as endowment funds: in 2008, the French government cancelled part of its debt in exchange of a capital endowment to FAPBM for a total amount of 13,325,000 euros. Such mechanisms allow governments to channel funds which initially had been intended to finance certain activities, to environmental endeavors.

- Sinking funds (*fonds d'amortissement*): in 2003, as a remission of debt from the government of Madagascar to the government of Germany through its bank KfW, the two entities agreed that the government of Madagascar would pay a total of 10.226 million euros to FAPBM, including 1.726 million euros as endowment and 8.5 million euros as sinking funds—to be spent within a fixed deadline. It was agreed that the government of Madagascar would pay the sinking funds in 20 annual installments of the same amount to FAPBM with the last payment happening in 2023. Because of defaults of payment, in 2015 the total reimbursement deadline was postponed to 2025 increasing the annual instalments to account for previous accumulated arrears. The sinking funds were and still are intended to support five PAs managed by MNP, namely Marojejy NP, Andringitra NP, Tsimanampesotse NP, Kirindy Mité NP, Ankarafantsika NP. FAPBM is providing MNP with the amount of the annual sinking funds installments even in the absence of payment by the Malagasy government to FAPBM.
- Revolving funds (*fonds renouvelable*): these are allocated as a loan to protected areas in order to implement specific projects/activities. These funds are replenished as PAs pay back the loans. This mechanism has never been implemented at FAPBM.
- Biodiversity offsets funds: Since 2019, FAPBM has acted as an intermediary for a biodiversity offset between an NGO managing the small Agnalazaha PA (IUCN cat. V, 2747 ha) to the north of the mining plant operating by QMM Madagascar Minerals, a Rio Tinto mining group subsidiary exploiting ilmenite in southeastern Madagascar. This offset program for an amount of US$ 300,000 finances serves to compensate the impacts of the mining activities.

*Supporting the conservation of the PAs and biodiversity by FAPBM*

Before being able to support PAs and biodiversity, FAPBM needs to cover its management costs estimated to be 15 % of its budget and which rank FAPBM amongst the most expensive trusts, considering that these costs are usually between 10 and 15 %[3]. With increased funding these management costs could decrease to a lower percentage.

Conventions

FAPBM provides grants based on conventions between institutions supporting conservation of PAs—such as MNP or other NGOs—and FAPBM. The foundation is

steadily supporting 36 PAs, including 22 PAs managed by MNP[4] (Montagne d'Ambre NP, Marojejy NP, Masoala NP, Mananara-Nord NP, Marotandrano SR, Ambatovaky SR, Zahamena NP, Ranomafana NP, Andringitra NP, Pic d'Ivohibe SR, Manombo SR, Befotaka/Midongy NP, Andohahela NP, Tsimanampetsotse NP, Mikea NP, Isalo NP, Kirindy Mité NP, Tsingy de Bemaraha NP, Baie de Baly NP, Ankarafantsika NP, Sahamalaza/Îles Radama NP, Tsaratanàna SNR (listed N to S, clockwise E to W, Fig 1). The support provided to MNP covers mainly the salaries for the management of these PAs. During the creation of any new PAs, it is mandatory that FAPBM compensate the resident communities living around the new PA being created.

Support for Capacity Building

In recent years a fund to support capacity building (*Fonds d'appui au Renforcement des Capacités*, FAR) has been created to help managers of newly created PAs, with funds that are mainly channeled via NGOs. More recently such funding was also made available to MNP.

[1] https://www.fapbm.org/categories-de-fonds-geres/

[2] P. Méral, G. Froger and F. Andriamahefazafy, "Financing protected areas in Madagascar: new methods," in E. Rodary and C. Aubertin, eds., *Protected Areas, Sustainable Land?* (Routledge, 2016): 105–120. https://doi.org/10.4324/9781315602646; P. Doinjashvili, P. Méral, and F. Andriamahefazafy, F., 2021. "Sustaining protected areas through conservation trust funds: a review," *International Journal of Sustainable Development & World Ecology*, 28, no 3 (2011): 193-202. And references therein. https://doi.org/10.1080/13504509.2020.1762257

[3] B. Spergel, and M. Wells, 2009. "Conservation Trust Funds as a Model for REDD+ National Financing," in A. Angelsen, ed., *Realising REDD+: National Strategy and Policy Option*, Center for International Forestry Research, Bogor, Indonesia, 2009: 75–84. Available online: https://pdfs.semanticscholar.org/7df9/6bd3888e959edab22734cbc7bcac2434e587.pdf#page=101

[4] https://www.fapbm.org/en/protected-areas-supported/
